# Pelvic bone marrow sparing intensity modulated radiotherapy reduces the incidence of the hematologic toxicity of patients with cervical cancer receiving concurrent chemoradiotherapy: a single-center prospective randomized controlled trial

**DOI:** 10.1186/s13014-020-01606-3

**Published:** 2020-07-29

**Authors:** Jin Huang, Fei Gu, Tianlong Ji, Jing Zhao, Guang Li

**Affiliations:** Department of radiotherapy, The First Affiliated Hospital of China Medical University, No. 155 Nanjing North Street, Shenyang, 110001 Liaoning China

**Keywords:** Pelvic bone marrow sparing intensity modulated radiotherapy (PBMS-IMRT), Cervical cancer, Hematological toxicity

## Abstract

**Purpose:**

To test the efficacy and feasibility of pelvic bone marrow sparing intensity modulated radiotherapy (PBMS-IMRT) in reducing grade 2 or higher hematological toxicity (HT2+) for patients with cervical cancer treated with concurrent chemoradiotherapy.

**Methods and materials:**

A total of 164 patients with Stage Ib2–IIIb cervical cancer were prospectively enrolled from March 2018 to March 2019 at a single center and were randomly allocated into the PBMS group or the control group. The control group received weekly cisplatin concurrently with IMRT, followed by intracavitary brachytherapy. The PBMS group additionally received PBM dose constraint. The dosimetric parameters of the pelvic bone (PB) and the subsites including hip bone (HIP) and lumbosacral spine (LSS) and the corresponding bone marrow were recorded. The endpoint of the trial was acute hematologic or gastrointestinal toxicity. Receiver operating characteristic curves were used to derive optimal dosimetric planning constraints.

**Results:**

Eighty-two patients in the PBMS group and 82 in the control group were enrolled for statistical analysis. The incidence of HT2+ in the PBMS group was 50.0%, significantly lower than the 69.5% incidence in the control group (*P =* 0.02). Patients with PB V40 ≥ 28% were more likely to experience HT2+ (OR = 2.85, *P* = 0.006), while the incidence of grade 2 or higher gastrointestinal toxicity (GT2+) events did not differ significantly between the two groups (*P* > 0.05). Dosimetric parameters of LSS showed stronger associations with HT2+ than other subsites. The patients with LSS V10 ≥ 87% and LSS mean ≥ 39 Gy were more likely to experience HT2+ (OR = 3.13, *P* = 0.001;OR = 3.03, *P* = 0.002, respectively).

**Conclusion:**

PBMS-IMRT reduced HT compared with IMRT alone. Efforts to maintain LSS V10 < 87%, LSS mean < 39 Gy and PB V40 < 28% simultaneously may reduce the risk of HT2 +.

**Trial registration:**

The trial was registered with Chinese clinical trial registry (ChiCTR1800015069).

## Introduction

Concurrent chemoradiotherapy (CRT) is a standard treatment that has been a great advance in the treatment of locoregionally-advanced cervical cancer. However, many side effects have appeared during the process of improving efficiency of CRT [1–4]. Studies have shown that many cervical cancer patients treated with CRT risk potential hematologic toxicity (HT), particularly grade 2 or higher leukopenia and neutropenia, with incidences of 30 to 45%, which could eventually lead to treatment breaks [5–7].

Bone marrow is the major hematopoietic organ consisting of active and inactive bone marrow. Approximately 51% of active bone marrow is located in the pelvis and lower spine [8, 9], regions which are included in the treatment volume with conventional pelvic radiotherapy. The unirradiated bone marrow can compensate for hematopoiesis even if the irradiated bone marrow is damaged during radiation therapy (RT) only. However, CRT causes damage to almost all hematopoietic stem cells (HSCs), as well as reducing the hematopoietic capacity of hematopoietic progenitor cells (HPCs), which can accelerate the incidence of hematotoxic events [8–13].

Various retrospective studies have demonstrated a correlation between RT dosimetric parameters and the incidence of acute HT [14–22]. Intensity modulated radiation therapy (IMRT) has a unique advantage in pelvic bone marrow sparing (PBMS) [23–25]. The multi-center prospective trial, INTERTECC-2, reported that IMRT can reduce acute hematologic and gastrointestinal toxicity compared to three-dimensional conformal radiotherapy [26]. Nonetheless, the feasibility of delineating pelvic bone as bone marrow, and the clinical outcomes for PBMS-IMRT still remain controversial. Therefore, we initiated a prospective randomized controlled trial to explore the benefits of PBMS-IMRT in acute toxicity and the challenges of dose planning.

## Methods and materials

### Study design and randomization

One hundred sixty-four eligible female patients with cervical carcinoma who were undergoing treatment with concurrent cisplatin and IMRT ± brachytherapy were prospectively recruited in this single-center prospective RCT study from March 2018 to March 2019.

Major inclusion criteria were: (1) Diagnosis of clinical Stage Ib2–IIIb cervical carcinoma with no previous history of chemotherapy or pelvic irradiation (according to the FIGO clinical stage of 2015 NCCN guidelines). Clinical stage was determined by gynecological examination, endometrial curettage, hysteroscopy, pelvic MRI, lung X-ray, neck and abdominal color Doppler ultrasound and other laboratory examinations. (2) Patients between 20 and 70 with ECOG score < 3 (as defined by Zubrod-ECOG-WHO) [27]. Exclusion criteria were: (1) Patients with blood-related diseases or occupational exposures such as paint, decoration, nail industry, etc.; (2) Patients who had been treated with extended-field radiation therapy; (3) Patients who were participating in other clinical trials at the same time. The study was approved by the institutional review board of the local institution. All patients were informed about the design and potential risk of the trial before the intervention and signed informed consents.

**Fig. 1:**
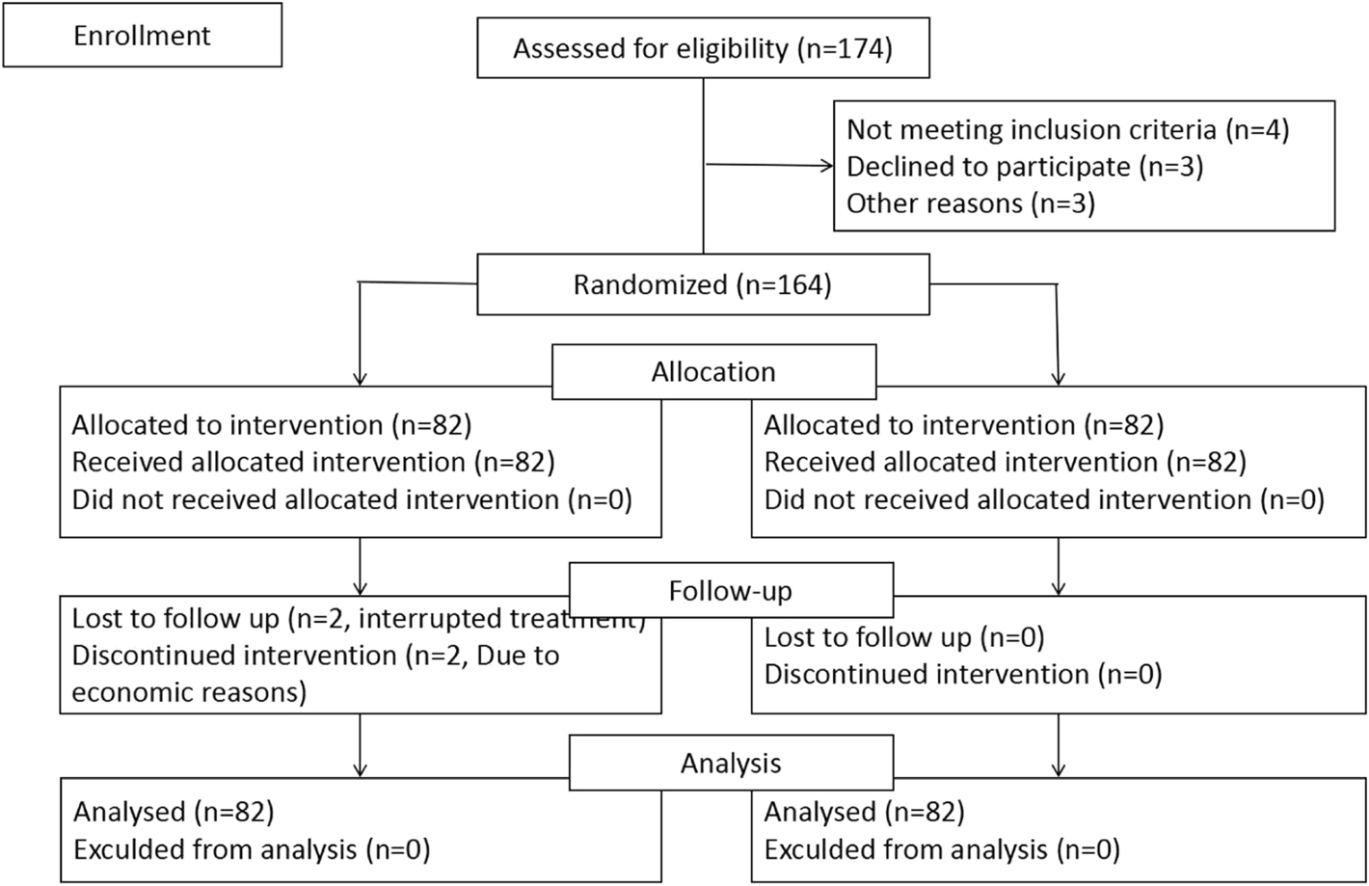
CONSORT flow diagram of patient selection and allocation

Eligible patients were enrolled on the basis of the CONSORT Statement Extension for Randomized Controlled Trials with allocation concealment, as shown in Fig. 1. A computer-generated randomization table was used to allocate the enrolled patients into the PBMS group (82 cases) or the control group (82 cases). The group-allocation information was blinded to both patients and doctors who participated in the study.

### Radiation simulation, planning and delivery

Patients were immobilized in the supine position and underwent a contrast-enhanced CT scan with a 5 mm slices from the L3 - L4 junction to 2 cm below the perineum. The image datasets were transferred to the Raystation planning system (Raysearch Radiation Oncology Systems). The clinical target volume (CTV) was divided into CTV-U, CTV-C, CTV-V, CTV-P and CTV-N determined by CT and MRI, which were defined as the uterus, cervix, vagina, paracancer tissue and the common iliac, internal iliac, external iliac, obturator and sacral lymph node region, respectively. The planning target volume (PTV) was defined by a uniform three-dimensional expansion using 10 to 20 mm around CTV-U and CTV-C, 10–15 mm around CTV-V and CTV-P, 7 mm around CTV-N. All patients had bladder filling and rectal emptying before radiotherapy.

IMRT plans were generated with 7–9 coplanar fields using a 6-MV X ray. The planned IMRT dose was 50.4 Gy in 1.8 Gy daily fractions. PTV planning constraints were to ensure that the PTV should receive 95% of the prescribed dose and that 107% of the prescribed dose should be restricted to ≤5% of the PTV. The bladder, rectum, and small bowel were contoured as the Organs at risk for all patients.

Patients treated definitively received 3D image-based brachytherapy with 4–5 fractions of 6–7 Gy. Treatment volumes and dose prescriptions based on GEC-ESTRO recommendations [28]. The mean dose to 90% of the PTV was required to achieve the prescribed dose. Brachytherapy was initiated no sooner than the fourth week of treatment and insertions were separated by a minimum of 48 h.

### Delineation of pelvic bone and marrow in PBMS-IMRT

**Fig. 2:**
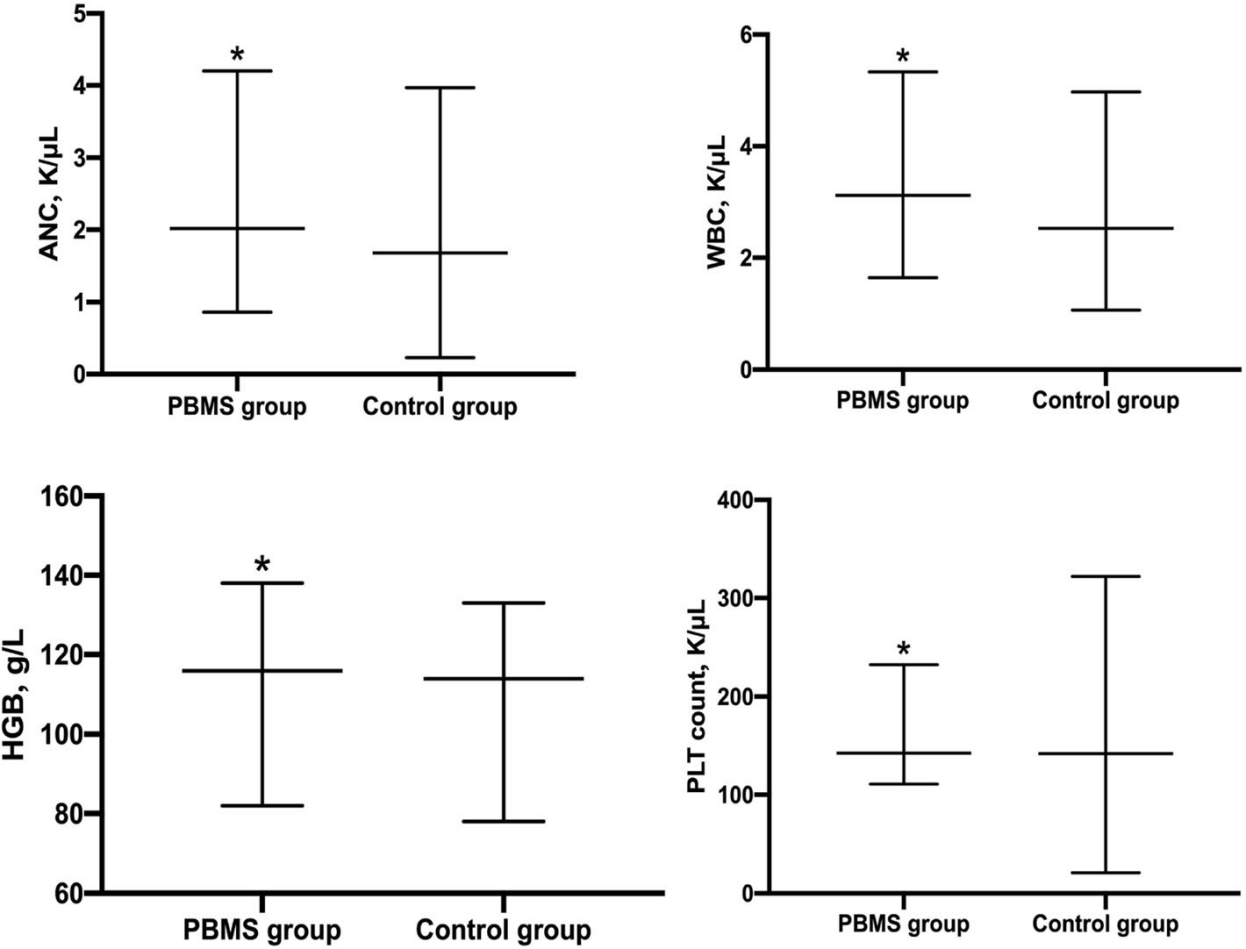
The longitudinal plots of the median blood counts including ANC (**a**), WBC (**b**), HGB (**c**), and platelet count (**d**). * Statistically significant. Abbreviations as in Tables 1

For each patient, the external contour of all bones within the pelvis was used as a surrogate for pelvic bone (PB) which was delineated on the window of a planning CT scan (window width: 800 Hu–2000 Hu; window level: 250 Hu–500 Hu). PB was further divided into the hip bone (HIP) and lumbosacral spine (LSS). HIP was the area from the iliac crests extending to the proximal femora, which consisted of the pubes, ischia, acetabula, and proximal femora. LSS was the area extending from the most superior vertebral body contained in the planning treatment volume (usually L5) inferiorly to the entire sacrum. Bone marrow was defined as the low-density regions within the corresponding bones. Pelvic bone marrow (PBM) was also divided into two subsites, namely HIP marrow and LSS marrow (Figs. 1s and 2s).

According to various retrospective studies, the volumes receiving above 10 Gy (V10) and the mean dose of LSS, as well as the volumes receiving above 10, 20, or 40 Gy (V10, V20, V40) and the mean dose of PB were correlated with acute HT in cervical cancer patients with CRT [15–20]. Therefore, PBMS-IMRT was applied in the PBMS group in order to maximize the constraints of the pelvic bone dose parameters in: LSS V10 < 85%, LSS mean < 30 Gy, PB V10 < 80%, PB V20 < 70%, PB V40 < 30%, and PB mean < 30 Gy. The control group patients were treated with IMRT alone.

**Fig. 3:**
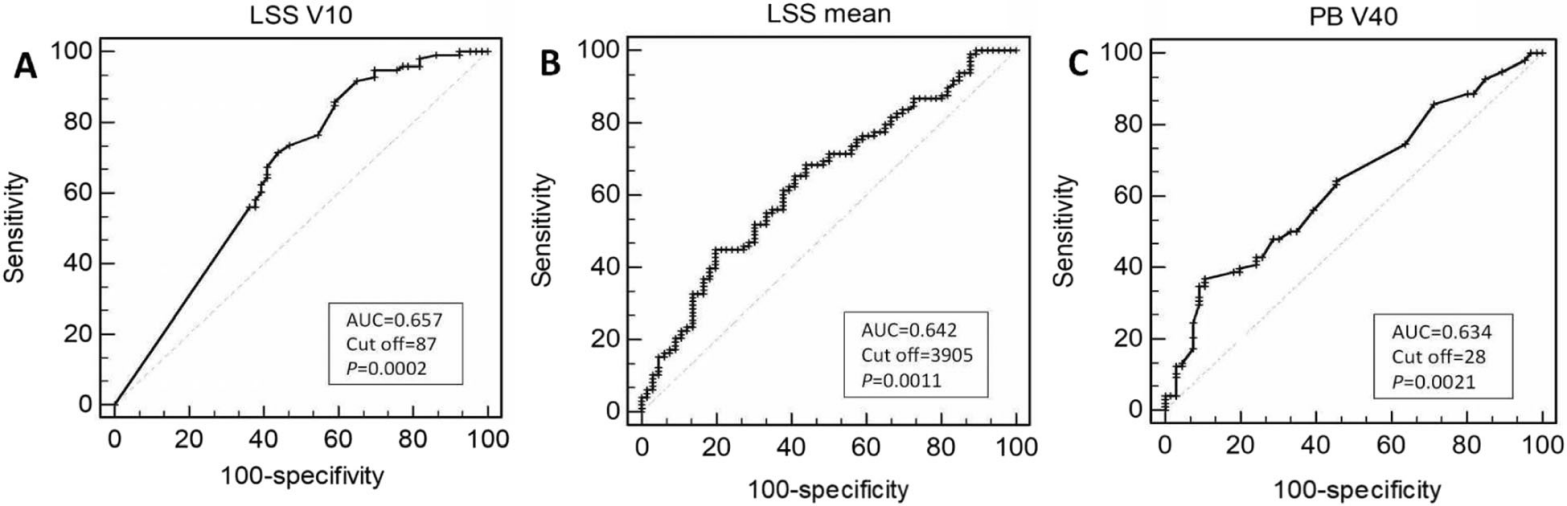
Receiver operating characteristic (ROC) curves for HT2+ as a function of LSS V10 (**a**), LSS Mean (**b**) and PB V40 (**c**)

After the completion of planning, dose volume histograms (DVHs) were then generated, and the parameters of PB and marrow were recorded (Figs. 3s and 4s). The treatment plan for all patients was established by two physicians and the radiotherapy plan was developed by the same medical physicist.

### Chemotherapy delivery and acute toxicity

Chemotherapy consisted of weekly cisplatin (40 mg/m^2^, maximal dose 70 mg) concurrently with IMRT ± brachytherapy. Cisplatin was held and appropriate symptomatic treatment was administered under the following conditions: white blood cell count (WBC) < 2 × 10^9^/L, absolute neutrophil count (ANC) < 1 × 10^9^/L, platelet (PLT) count < 50 × 10^9^/L, or creatinine clearance < 50 mL/min.

The primary endpoint was grade 2 or higher hematological toxicity (HT2+) during the treatment. The median follow-up time was the end of treatment. The blood counts monitoring and gastrointestinal symptoms of all patients were recorded weekly from the beginning to the end of CRT. And results were graded according to the Radiation Therapy Oncology Group acute radiation toxicity scoring criteria [29], with HT2+ or gastrointestinal toxicity (GT) of grade ≥ 2 (GT2+) noted as an event.

### Statistical analysis

Regarding the the sample size calculation and methods for power calculation, We set α and β to be 0.05 and 0.1, respectively. The incidence of HT2+ in control group was estimated to be 0.72 according to the pre-test in our center. The incidence of HT2+ in PBMS group was estimated to be 0.46 according to multiple retrospective studies. The sample size of two groups was estimated to be 74 by two-sided test.

Statistical analysis was performed using SPSS 24.0 software (IBM SPSS Statistics for Windows, Armonk, NY, USA). Body mass index (BMI, calculated as body weight (kg)/height (m)^2^) and dosimetric parameters were coded as continuous variables. Categorical variables included age (dichotomized by 60 years), pathology, comorbidity, ECOG score and clinical stage (dichotomized by IIIb). The enumeration data following a normal distribution are represented by mean ± standard deviation ($\overline{x}$ ± s). Categorical data are represented by the number of cases (n) and percentage (%). According to different data, Student’s *t*-test, the chi-square test or the Mann-Whitney U rank test were used to compare the differences in means and proportions. Univariate logistic regression was used to test the correlation between clinical and dosimetric parameters with HT2+. Multivariate logistic regression models controlling for clinical stage, ECOG score, BMI, age, pathology and comorbidity were then used to examine the effect of significant dosimetric parameters on HT2+. Due to large inter-variable correlations within the dosimetric variables, only one of the dosimetric variables and the clinical factors were included in pairs for each multivariable analysis. Receiver operating characteristic (ROC) curves were used to evaluate the value of dosimetric parameters for predicting hematologic toxicity. Area under curve (AUC) was used to evaluate the goodness of the curve model. The ROC curve was plotted using Medcal 15.0.

## Results

### Patient and treatment characteristics

Between March 2018 and March 2019, 164 patients consented to the study, of which two patients in the PBMS group were discontinued for financial reasons. Data of all patients who initiated protocol therapy were analyzed according to intention-to-treat. Our study showed no difference in the clinical parameters and the first full blood count between the studied groups (Table 1).

**Table 1:** Patient characteristics

Characteristic	PBMS group	Control group	*p*
Patients, n	82	82	
Age,years (s.d.)	53.7 (8.9)	53.8 (7.9)	0.91
Height,cm (s.d.)	159 (4.6)	160.4 (5.3)	0.07
Weight,kg (s.d.)	61.6 (8.1)	62.0 (8.3)	0.55
Body mass index, kg/m^2^ (s.d.)	24.4 (2.4)	24.1 (3.0)	0.74
Pathology, n (%)			0.72
Squamous cell carcinoma	79 (96.3)	77 (93.9)	
Adenocarcinoma	3 (3.7)	5 (6.1)	
Clinical stage, n (%)			0.83
Ib2	2 (2.4)	4 (4.9)	
IIa1	3 (3.7)	2 (2.4)	
IIa2	2 (2.4)	3 (3.7)	
IIb	29 (35.4)	32 (39.0)	
IIIb	46 (56.1)	41 (50.0)	
ECOG, n (%)			0.82
1	71 (86.6)	72 (87.8)	
2	11 (13.4)	10 (12.2)	
Original routine blood test (s.d.)			
WBC,*10^9/L	7.0 (2.4)	7.0 (3.2)	0.94
ANC,*10^9/L	4.8 (2.3)	4.7 (2.8)	0.87
HGB, g/L	127.8 (14.2)	125.4 (17.8)	0.27
PLT,*10^9/L	268.1 (60.3)	269.3 (81.4)	0.85
Comorbidity, n(%)	18 (22.0)	22 (26.8)	0.47

*Abbreviation*: *s.d.* standard deviation, *WBC* white blood cell count, *ANC* absolute neutrophil count, *HGB* hemoglobin, *PLT* platelet count

### Dosimetric parameters

The dosimetric parameters were significantly different between the PBMS group and the control group (*P <* 0.01). Descriptive statistics of radiation dose volume parameters are presented in Table 2.

**Table 2:** Descriptive statistics of dosimetric parameters

Parameter	PBMS group (s.d.)	Control group (s.d.)	*p*
LSS			
Volumn (cc)	384.7 (63.7)	383.2 (69.5)	0.68
V10%	83.1 (8.5)	99.6 (2.8)	< 0.01^a^
Mean dose (cGy)	2960.1 (309.8)	3982.2 (290.3)	< 0.01^a^
PB			
Volumn (cc)	1164.4 (116.8)	1136.6 (161.3)	0.59
V10%	80.0 (6.2)	94.6 (3.9)	< 0.01^a^
V20%	61.3 (8.1)	81.7 (5.4)	< 0.01^a^
V40%	21.0 (3.1)	29.3 (6.1)	< 0.01^a^
Mean dose (cGy)	2644.4 (181.6)	3218.4 (196.6)	< 0.01^a^
LSS Marrow			
Volumn (cc)	45.7 (10.4)	44.8 (11.8)	0.76
V10%	95.0 (8.0)	99.8 (1.9)	< 0.01^a^
V20%	84.6 (13.0)	99.3 (3.7)	< 0.01^a^
V40%	37.7 (10.4)	63.3 (19.9)	< 0.01^a^
Mean dose (cGy)	3420.4 (454.8)	4190.7 (357.8)	< 0.01^a^
PB Marrow			
Volumn (cc)	386.3 (61.5)	398.2 (71.9)	0.49
V10%	79.7 (7.7)	93.4 (4.8)	< 0.01^a^
V20%	64.0 (8.9)	77.6 (7.6)	< 0.01^a^
V40%	18.2 (3.8)	24.9 (8.8)	< 0.01^a^
Mean dose (cGy)	2583.0 (260.2)	3018.5 (241.4)	< 0.01^a^
HIP Marrow			
Volumn (cc)	357.9 (61.7)	360.5 (61.1)	0.87
V10%	78.1 (5.8)	92.6 (5.0)	< 0.01^a^
V20%	64.0 (9.2)	74.8 (9.4)	< 0.01^a^
V40%	16.6 (4.2)	19.7 (7.2)	< 0.01^a^
Mean dose (cGy)	2547.5 (207.4)	2888.1 (252.6)	< 0.01^a^

*Abbreviation*: *V10, V20, V30, V40* volume receiving ≥ 10, 20, 30, 40 Gy, *LSS* lumbosacral spine, *PB* pelvic bone, *s.d.* standard deviation. ^a^Statistically significant

### Chemotherapy characteristics

In the PBMS group, 40 patients completed all six cycles, 33 completed five cycles, and seven completed four cycles, one completed two cycles and one completed one cycle. In the control group, 37 patients completed all six cycles, 31 completed five cycles, and 14 completed four cycles Cumulatively. Some patients have withheld or postponed CRT for some reasons, as detailed in Table 1s. There was no significant difference in the total number of chemotherapy cycles between the two groups (*P* = 0.47). 17 patients received granulocyte-monocyte colony stimulating factors and 2 patients received platelet transfusions during treatment. No patients received red blood cell transfusions or recombinant human erythropoietin injection.

### Acute toxicity

All patients enrolled had full blood count tests weekly and none of the patient missed a blood sample. The incidence of HT2 + in the PBMS group was significantly lower than that in the control group (50% vs 69.5%) (Table 3). The median WBC, ANC, hemoglobin (HGB), and PLT count nadirs in the PBMS group were 3.2 × 10^^9^/L (range, 1.63–5.33), 2.02 × 10^^9^/L (range, 0.86–4.20), 116.10 g/L (range, 82.00–138.00), 142.5.00 × 10^^9^/L (range, 111.00–232.00). Meanwhile the median WBC, ANC, HGB, and PLT count nadirs in the control group were 2.53 × 10^^9^/L (range, 1.07–4.97), 1.68 × 10^^9^/L (range, 0.23–3.97), 114 g/L (range, 78.00–133.00), and 142 × 10^^9^/L (range, 21.00–322.00), with significant differences (*P* < 0.05) (Fig. 2). The GT2+ events did not differ significantly between the two groups (*P* > 0.05).

**Table 3:** Acute hematologic toxicity

Toxicity	PBMS group n (%)	Control group n (%*)*	*p*
HT2+	41 (50.0)	57 (69.5)	0.02^a^
Leukopenia			
Grade 0	13 (15.9)	8 (9.8)	< 0.01^a^
Grade 1	32 (39.0)	22 (26.8)	
Grade 2	35 (42.7)	37 (45.1)	
Grade 3	2 (2.4)	15 (18.3)	
Grade 4	0	0	
Neutropenia			
Grade 0	51 (62.2)	35 (42.7)	<0.01^a^
Grade 1	20 (24.4)	11 (13.4)	
Grade 2	9 (11.0)	30 (36.6)	
Grade 3	2 (2.4)	5 (6.1)	
Grade 4	0	1 (1.2)	
Anemia			
Grade 0	68 (82.9)	52 (63.4)	0.02^a^
Grade 1	10 (12.2)	19 (23.2)	
Grade 2	4 (4.9)	11 (13.4)	
Grade 3	0	0	
Grade 4	0	0	
Thrombocytopenia			
Grade 0	81 (98.8)	68 (82.9)	< 0.01^a^
Grade 1	1 (1.2)	10 (12.2)	
Grade 2	0	2 (2.4)	
Grade 3	0	1 (1.2)	
Grade 4	0	1 (1.2)	
GT2+	29 (35.4)	31 (37.8)	0.75
Grade 0	14 (17.1)	17 (20.7)	0.68
Grade 1	39 (47.6)	34 (41.5)	
Grade 2	29 (35.4)	31 (37.8)	
Grade 3	0	0	
Grade 4	0	0	

*Abbreviation*: *HT2+* hematologic toxicity of grade ≥ 2, *GT2+* gastrointestinal toxicity of grade ≥ 2

^a^Statistically significant

### Multivariate logistic regression analysis of factors associated with the development of HT2+

For PB, LSS V10, LSS mean, PB V40 were significantly associated with HT2+. For PBM, V10, V20, V40, mean of LSS marrow and PB marrow V40 were significantly associated with HT2+, correspondingly (*P* < 0.05). No dosimetric parameters of the hip bone were associated with HT2+ in this analysis (Table 4).

**Table 4:** Multivariate logistic regression analysis of factors associated with the development of grade ≥ 2 hematologic toxicity

Parameter	Odds ratio	*p*	95CI
**LSS**			
V10	1.07	< 0.01^a^	1.03,1.11
Mean dose	1.001	< 0.01^a^	1.000,1.002
**PB**			
V10	1.04	0.06	0.99,1.08
V20	1.03	0.06	0.99,1.05
V40	1.09	0.03^a^	1.03,1.16
Mean dose	1.001	0.07	1.000,1.002
**LSS MARROW**			
V10	1.15	< 0.01^a^	1.05,1.24
V20	1.06	< 0.01^a^	1.02,1.09
V40	1.02	0.01^a^	1.00,1.04
Mean dose	1.001	< 0.01^a^	1.000,1.002
**PB MARROW**			
V10	1.04	0.06	0.99,1.08
V20	1.03	0.06	0.99,1.05
V40	1.09	< 0.01^a^	1.03,1.16
Mean dose	1.001	0.07	1.000,1.002
**HIP MARROW**			
V10	1.03	0.08	0.99,1.07
V20	1.02	0.17	0.99,1.05
V40	1.06	0.06	0.99,1.13
Mean dose	1.001	0.06	1.000,1.002

Each dosimetric variable was combined with all clinical variables (clinical stage, ECOG score, BMI, age, pathology and comorbidity). Odds ratio correspond to 1% increase in factors approximately increased or decreased relative odds of HT2+. *CI* onfidence interval; other abbreviations as in Tables 2

^a^Statistically significant

### ROC curves

To identify optimal thresholds for dosimetric planning, we analyzed the ROC curves for HT2+ versus LSS V10, LSS mean and PB V40. The optimal LSS V10 cutoff indicated by the ROC curve was 87%. The sensitivity and specificity for this threshold were 71.4 and 56.1. The patients with LSS V10 ≥ 87% were more likely to experience HT2+ (OR = 3.13, 95%CI = 1.62–6.05, *P* = 0.001). The optimal cutoff of LSS mean was 39.05 Gy. The sensitivity and specificity for this threshold were 36.7 and 89.4. The patients with LSS mean ≥ 39 Gy were more likely to experience HT2+ (OR = 3.03, 95%CI = 1.49–6.17, *P* = 0.002). The optimal cutoff of PB V40 was 28%. The sensitivity and specificity for this threshold were 36.7 and 89.4. The patients with PB V40 ≥ 28% were more likely to experience HT2+ (OR = 2.85, 95%CI = 1.35–6.01, *P* = 0.006) (Fig. 3).

## Discussion

This is a prospective randomized controlled trial to investigate the association between HT and dosimetric parameters of PBM in patients with cervical carcinoma who were undergoing CRT. RTOG 0418 suggested that high dose irradiation was a high risk factor for acute myelosuppression [18]. In our trial we also found that V40 of PB and PB marrow were the dosimetric parameters most relevant to HT2+. In addition, the incidence of HT2+ was significantly decreased (50.0% vs 69.5%) with a reduction in the volume of PBM exposed to high dose radiation. Less HT in women receiving CRT may reduce the need for chemotherapy dose reductions, potentially improving the efficacy of RT by reducing the likelihood of treatment interruption. In our study, although there was no significant difference in the total number of chemotherapy cycles between the two groups (*P* = 0.47) due to the appropriate symptomatic treatment. However, 45 patients (54.88%) had one or two cycles held in control group while 37 patients (45.12%) in PBMS group.

Some retrospective studies and NTCP models have shown that low-dose radiation to the PBM is associated with HT events [15–17]. BM is extremely radiosensitive, with histopathological changes evident even with doses as low as 4 Gy [30]. In our trial, PBMS-IMRT also significantly reduced the volume of PBM that was illuminated at least in the low and moderate dose ranges. These dosimetric benefits may translate into less chronic HT, which will probably increase the tolerance of recurrent patients to chemotherapy.

Moreover, we found an observed association between the LSS subsite exposure and HT, although the LSS does account for approximately 10% of the whole-body bone marrow, and the irradiation volume and dose of the LSS was significantly higher than that of the iliac spine. Previous studies have inferred that PBMS could be actualized by restricting the dose to the PB alone [15, 17]. A study of rectal cancer by Bazan et al. suggested that constraints on the LSS alone (such as LSS-V10 < 85% or LSS-mean < 28 Gy) may suffice to prevent the incidence of HT [20]. However, by restricting the dose to the PB alone, a homogeneous dose is difficult to achieve due to the huge volume of the PB. By restricting the dose to the LSS alone, on the other hand, the high dose volume of pelvic bone irradiation could not be guaranteed; in addition exposure to the HIP may increase considering the anatomical location of other organs at risk (OARs) (bladder and small intestine). To conclude, simultaneous dose restrictions for PB and LSS subsites were necessary.

Another issue of concern associated with PBM as a constraint in the planning process is its potential impact on the dose optimization of the PBMS-IMRT treatment plans. Our standard treatment plans provided excellent coverage of the PTV, and simultaneously achieved considerable sparing of the other OARs. Our study also showed that the mean dose of LSS Marrow was higher than that of the bones, suggesting an inhomogeneous dose in the LSS area, due to the location of the LSS adjacent to the target volume (especially the sacral lymph nodes). The PTV of the sacral lymph node region was generally defined by encompassing the CTV with a 7-mm margin [31, 32]. Normally, this causes an overlapped area with the LSS, which is a crucial reason for challenges in meeting the dose constraints.

Another prospective study of RTOG 1203 showed that pelvic IMRT was associated with significantly less GT and urinary toxicity than standard RT [33]. However, the potential impact of PBMS on the dose of other OARs, especially the intestine, was particularly noteworthy. In our PBMS-IMRT treatment plans, although the small bowel volume irradiated to at least the prescription dose increased (14.19% vs. 13.07%), GT2+ events did not differ significantly between the two groups. Thus, significant PBMS could be achieved without compromising the intestinal dose. This result was similar to the findings of Lujan et al. [34].

Our study contained a few limitations: first, single-centered studies may lead to regional bias. Secondly, all patients in this study suffered from cervical cancer, so it was uncertain whether our findings were applicable to other pelvic tumors. Thirdly our study excluded patients with retroperitoneal lymph node metastasis due to the finite sample quantity. Yet such patients have higher potential for myelosuppression caused by extended-field irradiation. Moreover, to improve on our results, we are currently following up subjects to observe whether PBMS-IMRT could bring survival benefits.

## Conclusion

In summary, a significant clinical benefit was seen in HT with PBMS-IMRT with no additional impact on bowel function. These results have important implications for patients with cervical cancer who are receiving CRT. Furthermore, it would be more reliable for the application of the entire bones as a proxy for bone marrow. Efforts to constrict the dose of LSS and PB simultaneously will result in better homogeneity of the pelvic region. However further study is still warranted on the effects of long-term toxicity and survival.

## Supplementary information

**Additional file 1: Figure 1s.** Coronal section illustrating delineation of hip bone (blue) and lumbosacral spine (green).

**Additional file 2: Figure 2s.** (A) Axial section illustrating delineation of pelvic bone cotouring defined by using a computed tomography density-based autocontouring algorithm in bone window. (B) Axial section illustrating delineation of hip bone marrow (dark blue) and lumbosacral bone marrow (pink) by freehand.

**Additional file 3: Figure 3s.** Representative axial, sagittal and coronal section from IMRT plan in the control group (A) and the PBMS group (B).

**Additional file 4: Figure 4s.** Dose volume histogram for whole bone sub volumes in the control group(A), the PBMS group(B). Curves describe different irradiation area as PTV (red), LSS (green), HIP (blue), PB (orange), LSS Marrow (pink), HIP Marrow (dark blue) and PB Marrow (purple).

**Additional file 5: Table 1s.** Reasons for the chemotherapy withholding or postponing.

## Data Availability

All data generated or analyzed during this study are available from the corresponding author on reasonable request.
