# Supplementary figures and images for: Pelvic bone marrow sparing intensity modulated radiotherapy reduces the incidence of the hematologic toxicity of patients with cervical cancer receiving concurrent chemoradiotherapy: a single-center prospective randomized controlled trial
Source: Radiat Oncol. 2020 Jul 29;15:180. doi: 10.1186/s13014-020-01606-3 (PMC7389381; doi:10.1186/s13014-020-01606-3)

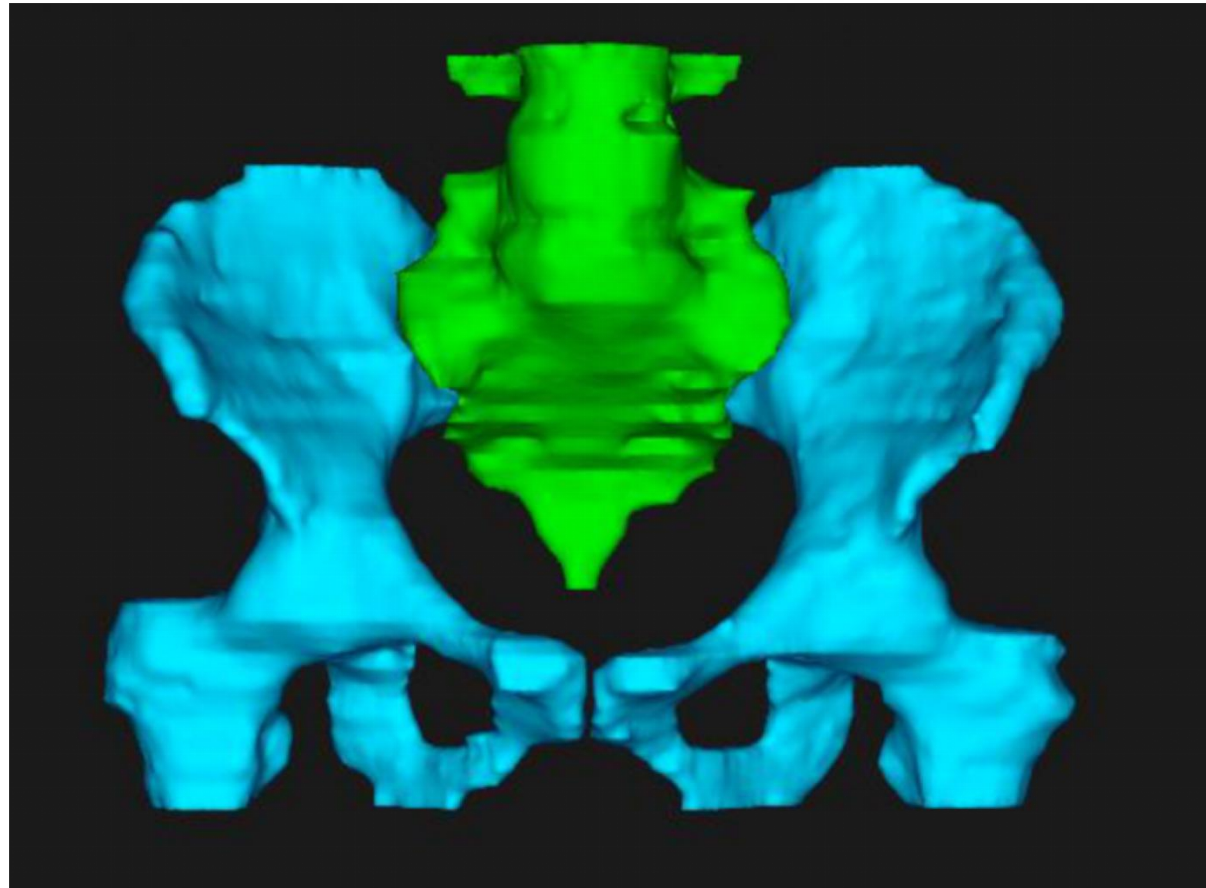

Supplement: Supplementary file 1 — Additional file 1: Figure 1s. Coronal section illustrating delineation of hip bone (blue) and lumbosacral spine (green). [file 13014_2020_1606_MOESM1_ESM.pdf]

A

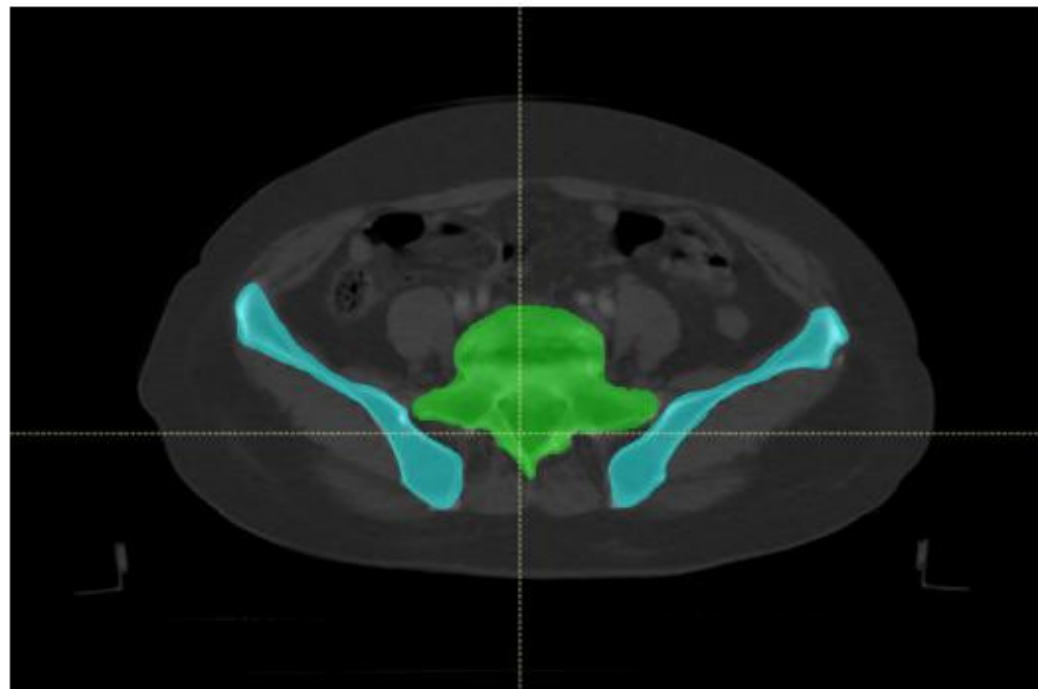

B

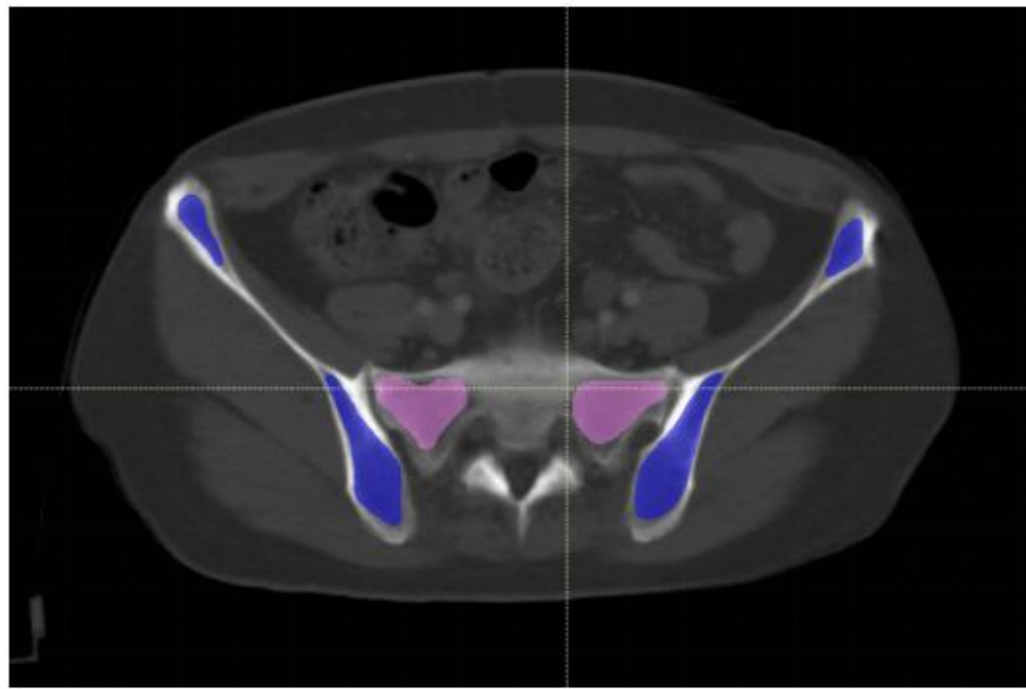

Supplement: Supplementary file 2 — Additional file 2: Figure 2s. (A) Axial section illustrating delineation of pelvic bone cotouring defined by using a computed tomography density-based autocontouring algorithm in bone window. (B) Axial section illustrating delineation of hip bone marrow (dark blue) and lumbosacral bone marrow (pink) by freehand. [file 13014_2020_1606_MOESM2_ESM.pdf]

A

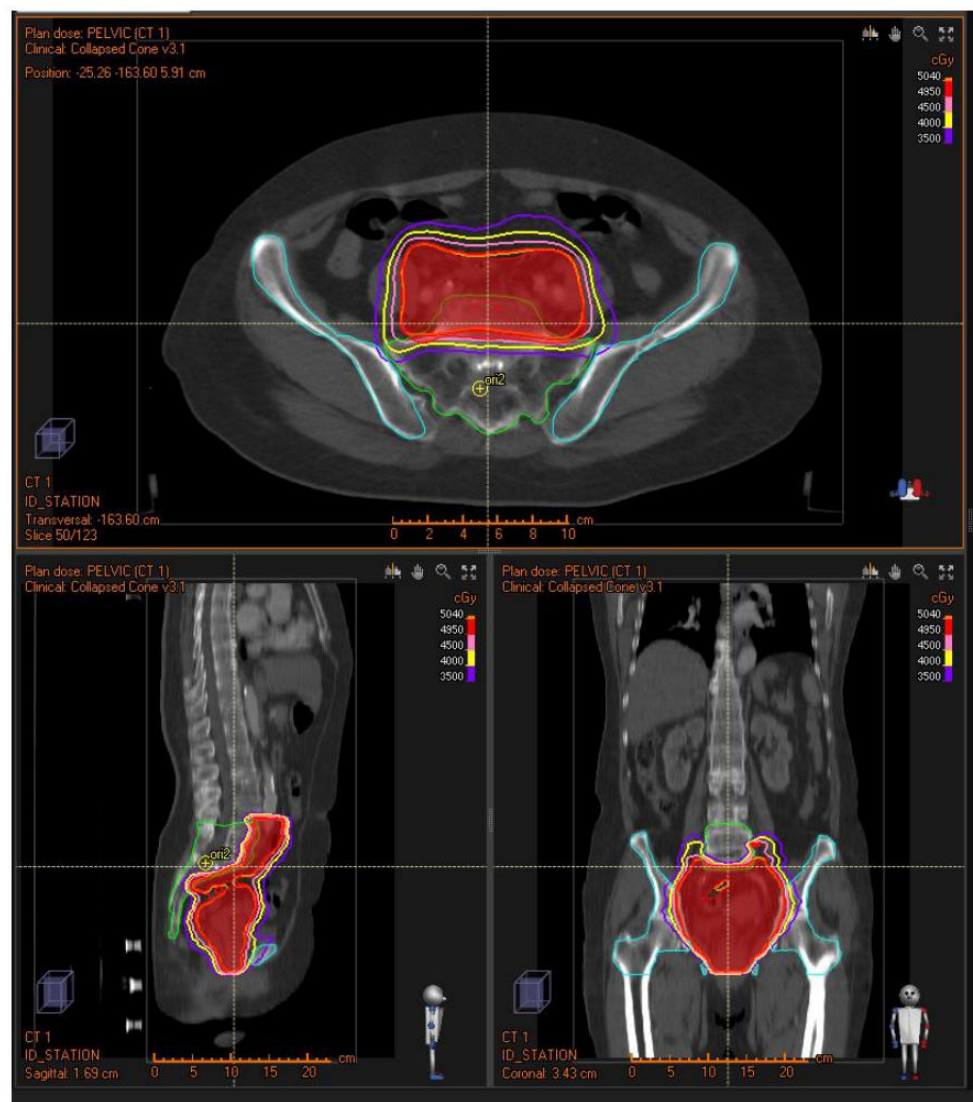

B

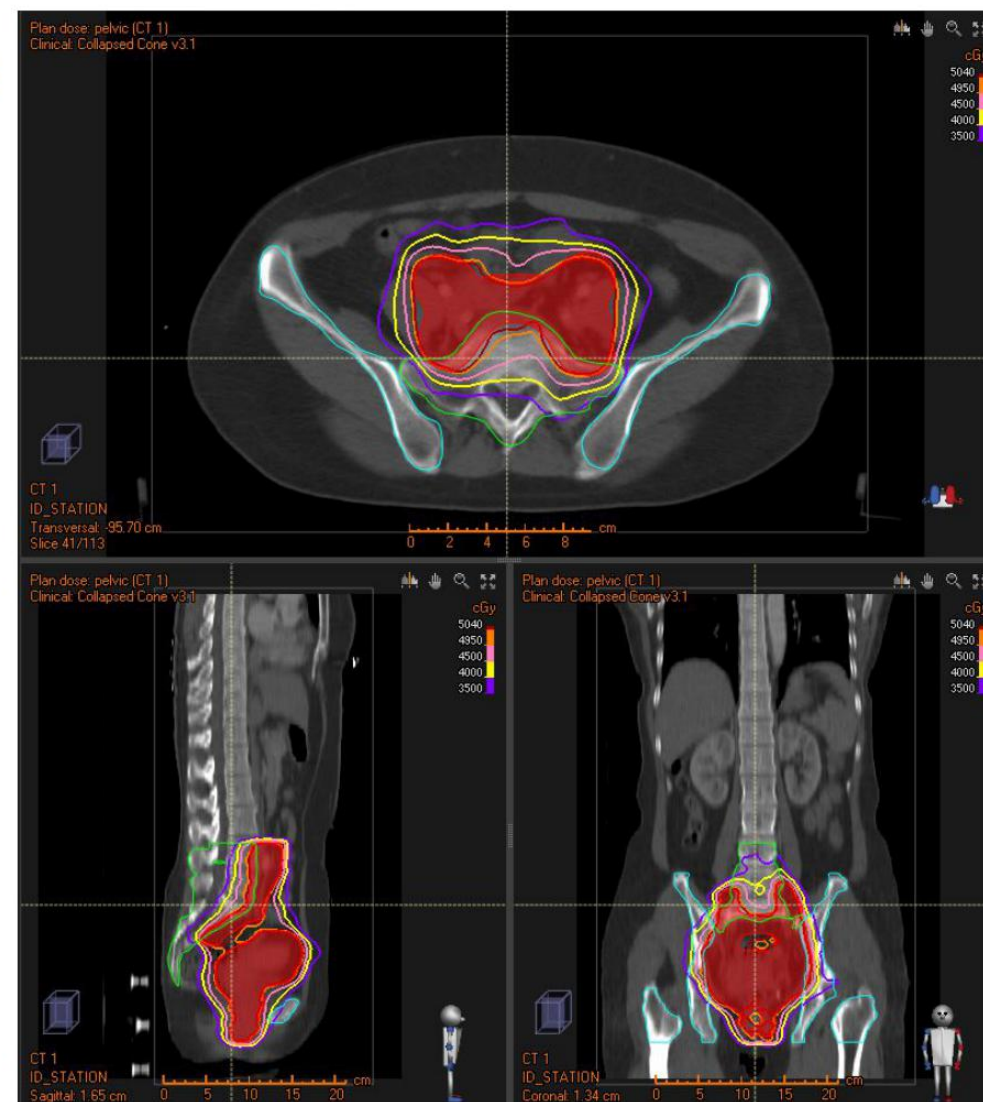

Supplement: Supplementary file 3 — Additional file 3: Figure 3s. Representative axial, sagittal and coronal section from IMRT plan in the control group (A) and the PBMS group (B). [file 13014_2020_1606_MOESM3_ESM.pdf]

A

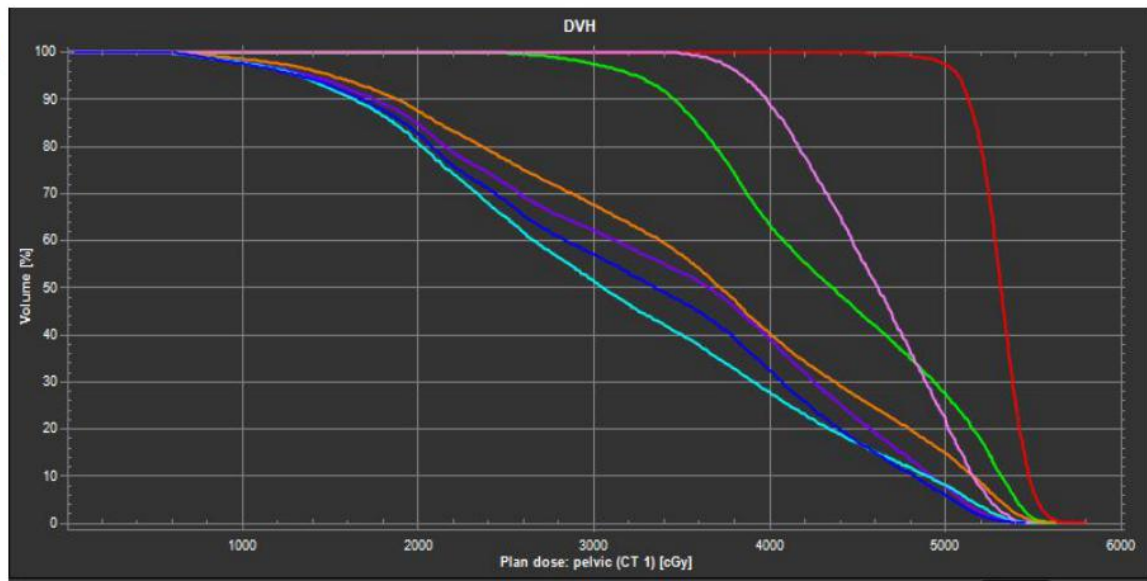

B

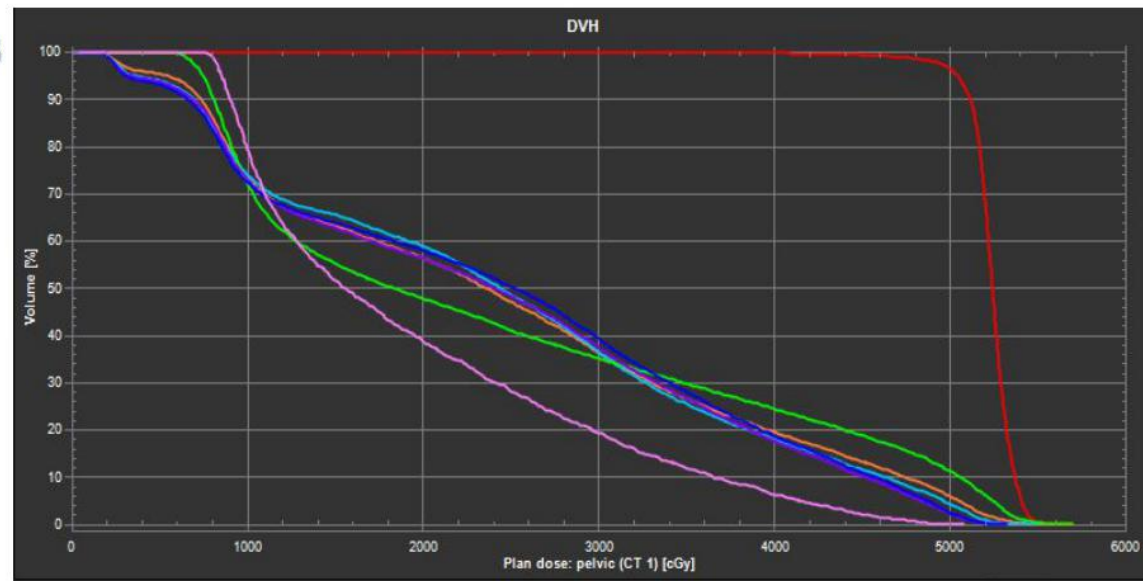

Supplement: Supplementary file 4 — Additional file 4: Figure 4s. Dose volume histogram for whole bone sub volumes in the control group(A), the PBMS group(B). Curves describe different irradiation area as PTV (red), LSS (green), HIP (blue), PB (orange), LSS Marrow (pink), HIP Marrow (dark blue) and PB Marrow (purple). [file 13014_2020_1606_MOESM4_ESM.pdf]
