# Supplementary material for: Pelvic bone marrow sparing intensity modulated radiotherapy reduces the incidence of the hematologic toxicity of patients with cervical cancer receiving concurrent chemoradiotherapy: a single-center prospective randomized controlled trial
Source: Radiat Oncol. 2020 Jul 29;15:180. doi: 10.1186/s13014-020-01606-3 (PMC7389381; doi:10.1186/s13014-020-01606-3)
Supplement: Supplementary file 5 — Additional file 5: Table 1s. Reasons for the chemotherapy withholding or postponing. [file 13014_2020_1606_MOESM5_ESM.docx]

| Table 2s. Reasons for the chemotherapy withhelding or postponing | |
| --- | --- |
| Reason | Condition |
| Hematological toxicity | WBC < 2 × 109/L |
|  | ANC < 1 × 109/L |
|  | PLT count < 50 × 109/L |
| Renal function | Creatinine clearance < 50 mL/min |
| Drug allergy | Eg. drug-induced rash |
| Chemotherapy side effects | Eg. severe nausea, vomiting |
| Poor physical condition | ECOG score > 2 |
| Other | Eg. refuse for psychological or economic reasons |

*Abbreviations:* WBC= white blood cell count; ANC= absolute neutrophil count; PLT= platelet count.
